# Nuclear Fission without Microtubules—a Return to the Past?

**DOI:** 10.1371/journal.pbio.1000515

**Published:** 2010-10-12

**Authors:** Kira Heller

**Affiliations:** Freelance Science Writer, Oakland, California, United States of America

**Figure pbio-1000515-g001:**
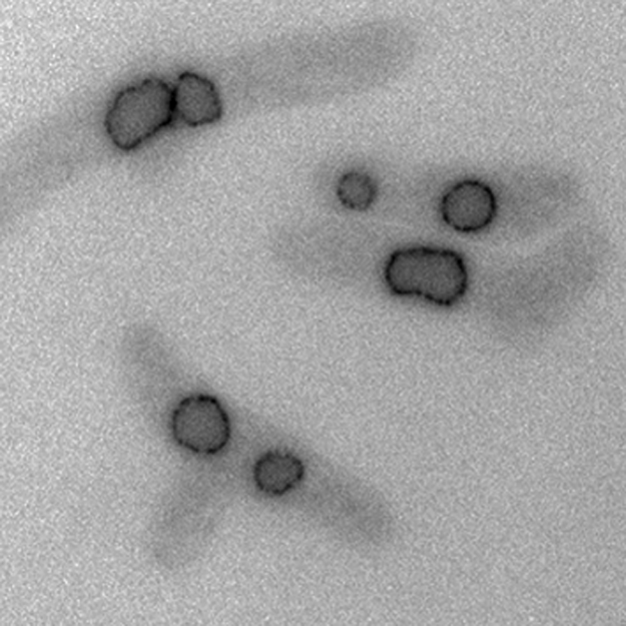
Though microtubule-based mitosis is a fundamental feature of cell division, new work in fission yeast provides evidence for a microtubule-independent nuclear division process in a eukaryotic cell, potentially uncovering an intermediate stage in the evolution of mitosis.

[Fig pbio-1000515-g001]One of the most familiar images in biology, even to high school students taking an introductory course, is that of newly duplicated chromosomes lined up along the center of a cell at the stage of cell division called metaphase. As the cell enters anaphase, microtubules, rod-like filaments that comprise the mitotic spindle, pull the chromosomes away from one another to opposite sides of the cell. The process is then completed when the mother cell pinches apart, producing two daughter cells, each with a full chromosomal complement. Cell division is subject to rigorous quality control in the form of checkpoints. One such checkpoint occurs during metaphase and ensures that all is well with the mitotic spindle, and that the microtubules are securely attached at one end to the kinetochore, an assemblage of proteins stuck to the center of each chromosome, and at the other to one of the two microtubule-organizing structures (“spindle pole bodies” in yeast) on opposite ends of the cell.

Not surprisingly then, spindle microtubules play a fundamental role in chromosome segregation; cells that lack microtubules are unable to pass the mitotic checkpoint and continue on to cell division. However, Stefania Castagnetti, Snezhana Oiferendo, and Paul Nurse (this issue of *PLoS Biology*) have made an unexpected observation in the fission yeast *Schizosaccharomyces pombe*, which undergoes a special form of mitosis, called closed mitosis, during which the nuclear membrane remains intact and the mitotic spindle elongates between two spindle pole bodies embedded in the nuclear envelope. Using a microtubule-depolymerizing drug (microtubules are made of protein subunits called tubulin) to prevent spindle formation and *S. pombe* mutants incapable of forming microtubules, the authors found an alternative form of nuclear division occurring in the absence of microtubules. This variation, which they call nuclear fission, could produce two clusters of segregated chromosomes, each encapsulated within a nuclear membrane.

To further investigate the conditions in which nuclear fission can occur, the authors used a variety of genetic tools and protein markers. They found that, unlike normal mitosis, nuclear fission was not under spindle checkpoint control; even though the absence of microtubules activated the spindle checkpoint, mitosis was only slightly delayed, and the nucleus proceeded to divide. They also determined that separation of sister chromatids (identical strands of a replicated chromosome connected by a structure called the centromere) was required for nuclear fission to occur, and that the centromeres of chromosomes remained clustered with the spindle pole bodies during nuclear fission. Using a drug that depolymerizes the cytoskeletal protein actin, they found that nuclear fission requires filamentous actin. Actin structures may be involved in the nuclear membrane redistribution that occurs in anaphase, and rapid redistribution facilitated by actin could be responsible for the ruffled appearance that the nuclear membrane acquires during fission.

Taking all of their results into consideration, the authors suggest a possible mechanism for this microtubule-independent cell division process: at the beginning of mitosis, rather than lining up at the cell center, the sister chromatids, connected by their centromeres, remain associated with the two spindle pole bodies. The spindle pole bodies then slowly move apart within the nuclear membrane, and as the sister chromatids lose cohesion with one another, they are pulled apart by their connection to the spindle pole bodies. Subsequent membrane expansion associated with lipid biosynthesis during mitosis and deformation of the nuclear envelope around the separated chromosomes then leads to the formation of distinct nuclear bodies.

Although not as gracefully orchestrated or efficient as conventional mitosis in fission yeast or other eukaryotes, this alternative form of nuclear division may have evolutionary implications. Unlike in eukaryotes, bacterial and archaeal cells segregate their DNA without a mitotic spindle. Instead, bacterial chromosome segregation is driven by polymerization of an actin-like protein. By showing that *S. pombe* can undergo nuclear fission without microtubules, Nurse and his colleagues may have peeled back the mitotic spindle overlay to provide a glimpse of an evolutionarily distant intermediate process in which cell division occurred when replicated sister chromosomes remained attached to centrosomes or spindle pole bodies at the nuclear envelope and were segregated as these structures moved apart. Further studies will reveal whether processes similar to nuclear fission in *S. pombe* occur in other eukaryotes, indicating that it could be an ancestral means of cell division prior to the evolution of the mitotic spindle.

**Castagnetti S, Oliferenko S, Nurse P (2010) Fission yeast cells undergo nuclear division in the absence of spindle microtubules. doi10.1371/journal.pbio.1000512**

